# Healthcare-associated infections (HAIs) during the coronavirus disease 2019 (COVID-19) pandemic: A time-series analysis

**DOI:** 10.1017/ash.2022.361

**Published:** 2023-01-17

**Authors:** John M. Sahrmann, Katelin B. Nickel, Dustin Stwalley, Erik R. Dubberke, Patrick G. Lyons, Andrew P. Michelson, Kathleen M. McMullen, Sumanth Gandra, Margaret A. Olsen, Jennie H. Kwon, Jason P. Burnham

**Affiliations:** 1 Division of Infectious Diseases, Washington University in St. Louis School of Medicine, St. Louis, Missouri; 2 Division of Pulmonary and Critical Care Medicine, Washington University in St. Louis School of Medicine, St. Louis, Missouri; 3 Infection Prevention, Mercy Hospital St. Louis, St. Louis, Missouri

## Abstract

**Objective:**

To use interrupted time-series analyses to investigate the impact of the coronavirus disease 2019 (COVID-19) pandemic on healthcare-associated infections (HAIs). We hypothesized that the pandemic would be associated with higher rates of HAIs after adjustment for confounders.

**Design:**

We conducted a cross-sectional study of HAIs in 3 hospitals in Missouri from January 1, 2017, through August 31, 2020, using interrupted time-series analysis with 2 counterfactual scenarios.

**Setting:**

The study was conducted at 1 large quaternary-care referral hospital and 2 community hospitals.

**Participants:**

All adults ≥18 years of age hospitalized at a study hospital for ≥48 hours were included in the study.

**Results:**

In total, 254,792 admissions for ≥48 hours occurred during the study period. The average age of these patients was 57.6 (±19.0) years, and 141,107 (55.6%) were female. At hospital 1, 78 CLABSIs, 33 CAUTIs, and 88 VAEs were documented during the pandemic period. Hospital 2 had 13 CLABSIs, 6 CAUTIs, and 17 VAEs. Hospital 3 recorded 11 CLABSIs, 8 CAUTIs, and 11 VAEs. Point estimates for hypothetical excess HAIs suggested an increase in all infection types across facilities, except for CLABSIs and CAUTIs at hospital 1 under the “no pandemic” scenario.

**Conclusions:**

The COVID-19 era was associated with increases in CLABSIs, CAUTIs, and VAEs at 3 hospitals in Missouri, with variations in significance by hospital and infection type. Continued vigilance in maintaining optimal infection prevention practices to minimize HAIs is warranted.

During the coronavirus disease 2019 (COVID-19) pandemic, secondary bacterial infections have been seen in patients with COVID-19.^1–6^ National Healthcare Safety Network (NHSN) data have demonstrated higher rates of central-line–associated bloodstream infection (CLABSI), catheter-associated urinary tract infection (CAUTI), and ventilator-associated events (VAEs), compared to the prepandemic period and as a function of COVID-19 case load. However, these studies have lacked correlation between patient-level risk factors and development of infections during hospitalization.^1,4^

Interrupted time-series analyses are used in public health research to estimate changes in a measured outcome before and after an intervention.^7,8^ However, consequences of natural phenomena can also be studied given a well-defined change point.^9^ We used interrupted time-series analysis to investigate the impact of the COVID-19 pandemic on healthcare-associated infections (HAIs) at 3 hospitals in St. Louis, Missouri. Time series of HAIs were compared before and after March 2020, when COVID-19 cases were first detected regionally. Our goal was to determine whether the pandemic was associated with higher HAI rates after adjusting for confounding variables. Such an increase has implications for infection prevention, antimicrobial stewardship, and other processes of care.

## Methods

### Data sources

This retrospective observational study was approved by the Washington University School of Medicine Institutional Review Board with a waiver of informed consent. Patient-level data were obtained for hospitalizations during January 1, 2017, through August 31, 2020, from the BJC HealthCare Clinical Data Repository. Data were obtained from 3 hospitals in the BJC Healthcare System that treated most COVID-19 patients, including 1 large quaternary-care referral hospital and 2 community hospitals. Infection control surveillance data reported to NHSN were collected from each of the 3 hospitals. CLABSIs, CAUTIs, and VAEs were defined according to CDC definitions, adjudicated by each hospital’s team of infection preventionists. Counts of CLABSIs, CAUTIs, and VAEs, as well as the number of central-line, urinary catheter, and ventilator days were aggregated monthly for each facility. The prepandemic period was defined as January 1, 2017–February 29, 2020. The pandemic period was defined as March 1, 2020–August 31, 2020. Medical record review was performed for admissions with a COVID-19 ICD-10-CM diagnosis code without a positive laboratory result to confirm a clinical diagnosis of severe acute respiratory coronavirus virus 2 (SARS-CoV-2) infection.

### Covariates

Inpatient stays at each facility of >48 hours duration were used to define monthly admission counts, total days admitted, and average length of stay. In addition, *International Classification of Diseases, Tenth Revision, Clinical Modification* (ICD-10-CM) diagnosis codes were used to compute the Elixhauser comorbidity index for each admission, and these data were averaged by facility and month.^10^ The number of beds at each facility was treated as a constant throughout the study period.

### Statistical analysis

Monthly CLABSI, CAUTI, and VAE counts were modeled separately using generalized linear models with a Poisson distribution and log link. Data from all facilities across all months were pooled into a single model for each type of HAI, and the monthly days of exposure (central-line days for CLABSIs, urinary catheter days for CAUTIs, and ventilator days for VAEs) were included as covariates to account for differences among hospitals. Mixed-effects models with hospital as a random effect were performed, but the estimated variance component was not significantly different from zero for any HAI. The effects of COVID-19 were modeled using an indicator variable taking the value zero prior to March 2020 and one thereafter to allow for change in the level of each HAI, and as a linear term expressed as the rate of COVID-19 cases per 10,000 inpatient days to allow for change in the trend.^8^ Other covariates included monthly admission counts, monthly mean length of stay, monthly mean in-hospital Elixhauser comorbidity index, and number of beds, as described above. Harmonic terms were used to account for secular trends unrelated to the pandemic.^11^

Model fit was assessed graphically by examining time series of residuals stratified by facility, plots of residuals and linear predictors, and plots of the estimated mean–variance relationship implied by each model. An estimate of the dispersion parameter was also computed.^12^ Quasi-Poisson and negative binomial models were fitted to check for improvement in the mean–variance relationship.

The excess number of each HAI attributable to the COVID-19 pandemic (ie, the difference between predicted vs actual HAIs during the COVID-19 pandemic) was estimated using 2 counterfactual scenarios. In the first (ie, “no COVID-19 cases”), the values of all monthly covariates were left unchanged except the rate of COVID-19 cases per 10,000 inpatient days, which was set to zero across all pandemic period months. Predicted numbers of each HAI during the pandemic period were then generated from the original fitted model and compared to the actual number of infections. The intention of this scenario was to allow for pandemic-related disruptions to hospital operations while exploring how the number of infections would have differed had no COVID-19 cases been seen by any facility.

Scenario 2 envisioned a situation in which the pandemic never happened (ie, “no pandemic”). First, alternative models were fit using prepandemic data with all covariates from the original models retained except those designed to capture the effects of COVID-19, that is, all covariates were retained except for the pandemic indicator variable and the rate of COVID-19 cases per 10,000 inpatient days. Then, hypothetical values were generated for each pandemic period monthly covariate by forecasting based on the prepandemic time-series associated with each facility.^13^ Forecasts were computed using seasonal ARIMA models, with the parameters of each model chosen using the procedure described by Hyndman and Khandakar^14^ and implemented by Hyndman and Athanasopoulos.^15^ Finally, the facility-specific forecasts were incorporated into the alternative models, generating predicted values and 95% confidence intervals for each HAI during the pandemic period, which were compared to the actual number of infections.

As a secondary analysis, total attributable HAIs under both counterfactual scenarios were re-estimated after excluding the first 2 months of the pandemic period (March and April 2020) in an attempt to isolate the effect of COVID-19 cases from the general disruption in hospital operations that occurred at the start of the pandemic (eg, personal protective equipment shortages, changes in admission rates, hospital case mix, canceling of elective surgeries).

Data management was performed using SAS version 9.4 software (SAS Institute, Cary, NC), and analyses were conducted using R version 4.1.2 software (R Foundation for Statistical Computing, Vienna, Austria).

## Results

A comparison of facility characteristics during the prepandemic and pandemic periods is provided in Table 1. Hospital 1 had the highest average number of monthly HAIs of each type but also had much higher levels of exposure (ie, higher numbers of central-line, urinary catheter, and ventilator days) than the 2 community hospitals. In the aggregate, the start of the pandemic was associated with modest changes in hospital operations; differences tended to be larger at hospital 3 (eg, total number of admissions and patient case mix). All hospitals experienced slight increases in average length of stay and more pronounced increases in the severity of illness of newly admitted patients as measured by the Elixhauser mortality comorbidity index (Table 1).

**Table 1.** HAI Monthly Averages by Hospital Before and During the COVID-19 Pandemic

Variable	Hospital 1 (Jan 2017– Feb 2020)^a^	Hospital 1 (Mar–Aug 2020)^a^	Hospital 2 (Jan 2017– Feb 2020)^a^	Hospital 2 (Mar–Aug 2020)^a^	Hospital 3 (Jan 2017– Feb 2020)^a^	Hospital 3 (Mar–Aug 2020)^a^
CLABSIs	13 (4)	13 (6)	1 (1)	2 (3)	1 (1)	2 (1)
Central-line days	10,674 (527)	10,374 (1060)	1,262 (146)	1,235 (123)	1,621 (134)	1,395 (285)
CAUTIs	8 (3)	6 (2)	1 (1)	1 (1)	1 (1)	1 (1)
Urinary catheter days	6,234 (216)	6,109 (703)	772 (139)	780 (145)	1,379 (156)	1,235 (272)
VAEs	8 (3)	15 (4)	2 (1)	3 (3)	1 (1)	2 (2)
Ventilator days	1,707 (159)	1,811 (177)	296 (91)	263 (37)	234 (59)	330 (77)
COVID-19 cases	N/A	131 (59)	N/A	82 (41)	N/A	52 (24)
Inpatient days	30,177 (2284)	29,017 (2913)	6,464 (494)	6,209 (587)	8,935 (715)	7,781 (1332)
Admissions	3,564 (253)	3,356 (413)	906 (70)	829 (115)	1,427 (94)	1,210 (218)
Length of stay, d	8.5 (0.2)	8.7 (0.5)	7.1 (0.3)	7.6 (0.8)	6.3 (0.2)	6.4 (0.1)
Elixhauser in-hospital comorbidity index	9.0 (0.5)	10.6 (0.7)	10.6 (0.6)	12.4 (1.2)	8.1 (0.6)	8.9 (0.4)
Beds	1,266	1,266	220	220	449	449

Note. HAI, hospital-acquired infection; CLABSI, central-line–associated bloodstream infection; CAUTI, catheter-associated urinary tract infection; VAE, ventilator-associated event.

a Numbers are average (standard deviation) of monthly counts unless otherwise specified.

**Fig. 1.**
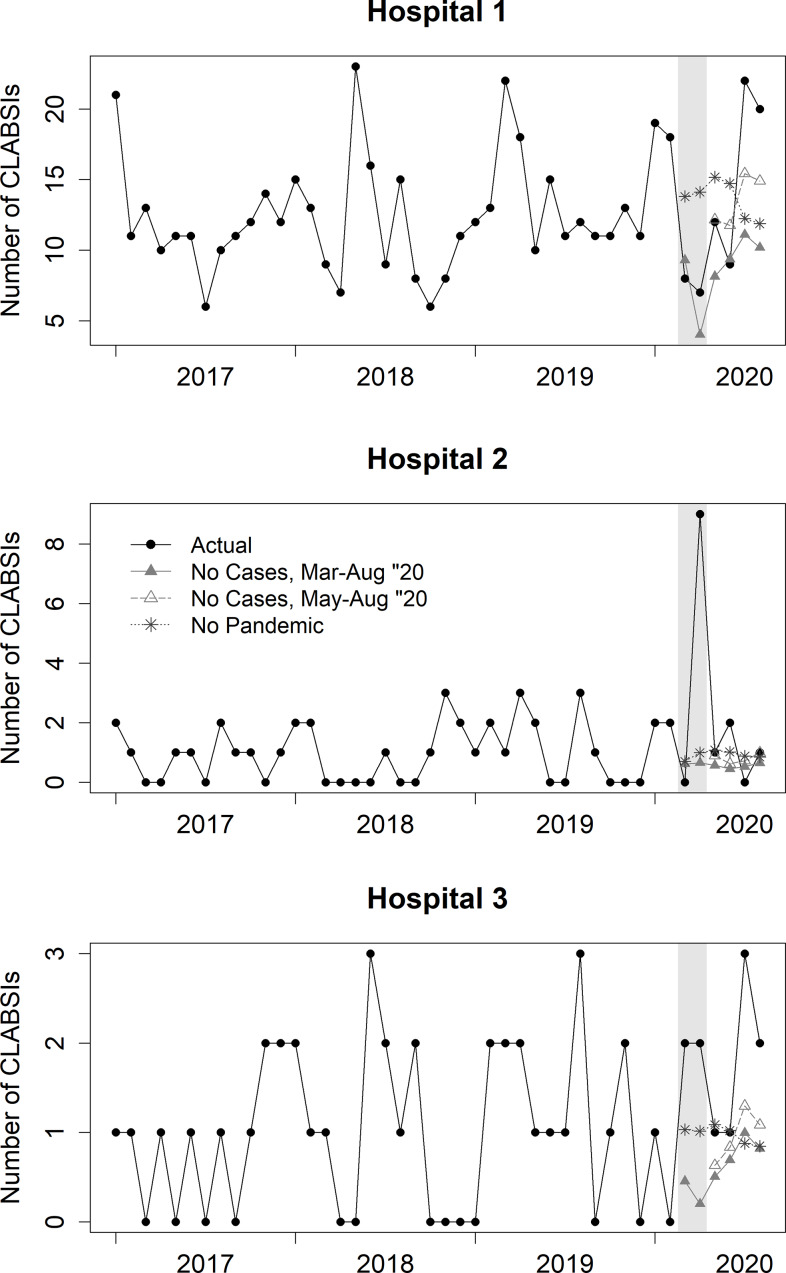
Estimating central-line–associated bloodstream infections (CLABSIs) by hospital during the COVID-19 pandemic under actual and counterfactual scenarios.^a^ Time series data in black are raw data, the remaining lines are predicted values and have been adjusted for covariates. The legend for hospital 2 applies to all plots. ^a^Grey area for March–April 2020 was not included in the secondary analysis.

The time series of the actual number of HAIs as well as the predicted number of HAIs under each counterfactual scenario are displayed in Figure 1 and Supplementary Figures 1 and 2, with the predicted values adjusted for covariates. Infection control specialists at hospital 1 documented 78 CLABSIs during the pandemic period as well as 33 CAUTIs, and 88 VAEs. During the same period, hospital 2 documented 13 CLABSIs, 6 CAUTIs, and 17 VAEs. Hospital 3 documented 11 CLABSIs, 8 CAUTIs, and 11 VAEs.

**Fig. 2.**
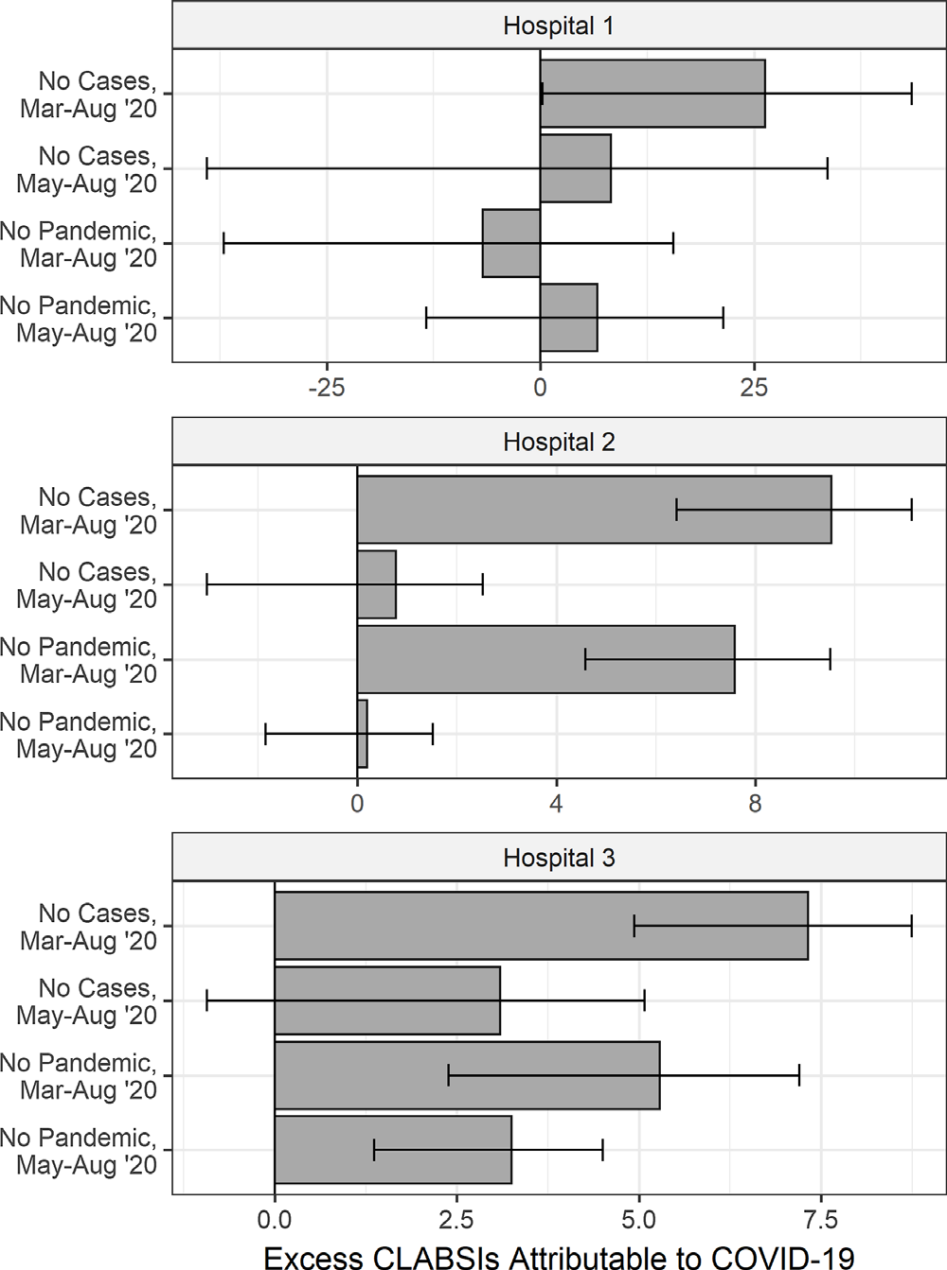
Estimating total excess central-line–associated bloodstream infections (CLABSIs) by hospital during the COVID-19 pandemic with counterfactual scenarios.

Total excess HAIs at each facility attributable to the pandemic are shown in Figure 2, and Supplementary Figures 3 and 4, adjusted for monthly admission counts, mean length of stay, mean Elixhauser comorbidity index, number of hospital beds, time trends unrelated to the pandemic, and central-line, urinary catheter, and ventilator days for the corresponding infection. Confidence intervals for hospital 1 included zero under all scenarios, excepting CLABSIs under the “no cases” scenario. At hospital 2, excess CLABSI estimates excluded zero under both scenarios: no cases, 9.5 (95% CI, 6.4–11.1) and no pandemic, 7.6 (95% CI, 4.6–9.5). Excess CAUTIs at hospital 2 were also significantly different from zero under all scenarios: no cases, 4.2 (95% CI, 1.4–5.3) and no pandemic, 4.6 (95% CI, 2.9–5.3). Also, at hospital 2, VAEs were significant under the “no pandemic” scenario: 8.2 (95% CI, 3.5–11.3). At hospital 3, CLABSIs were significantly higher in both scenarios: no cases, 7.3 (95% CI, 4.9–8.7) and no pandemic, 5.3 (95% CI, 2.4–7.2). Excess CAUTIs were not significantly different from zero under either scenario, and excess VAEs excluded zero under the “no pandemic” scenario at 3.8 (95% CI, 0.1–6.3).

After excluding March and April 2020 from the pandemic period (Fig. 1 and Supplementary Figs. 1 and 2), hospital 1 had 63 CLABSIs, 25 CAUTIs, and 59 VAEs. Hospital 2 had 4 CLABSIs, 3 CAUTIs, and 11 VAEs, and hospital 3 had 7 CLABSIs, 7 CAUTIs, and 7 VAEs. Estimates of excess HAIs were generally similar to those in the primary analysis (Fig. 2, Supplementary Figs. 3 and 4). Hospital 1 had increased case numbers of all infection types across scenarios, but these were not significant. At hospital 2, CLABSI estimates increased but not significantly. At hospital 2, excess CAUTIs remained significantly greater than zero under all scenarios: no cases, 2.3 (95% CI, 1.1–2.8) and no pandemic, 2.3 (95% CI, 1.2–2.7). At hospital 2,VAEs were still significant under the “no pandemic” scenario at 5.2 (95% CI, 2.1–7.3). At hospital 3, CLABSIs were no longer significantly different from zero under “no cases” but remained significantly higher under “no pandemic” at 3.2 (95% CI, 1.4–4.5). In contrast to the primary analysis, CAUTIs were significantly higher under both scenarios at hospital 3: no cases, 4.5 (95% CI, 0.7–6.0); no pandemic, 2.6 (95% CI, 0.1–4.2). The estimate of VAEs under the “no pandemic” scenario was no longer significantly different from zero (Supplementary Table 1).

## Discussion

Using interrupted time-series analysis, we detected increases in CLABSIs, CAUTIs, and VAEs during the early part of the COVID-19 pandemic under a variety of hypothetical scenarios, though statistical significance was not reached at each hospital. HAI rates (specifically CLABSIs, CAUTIs, and VAEs)compared to the counterfactual scenarios, were higher during the pandemic period, consistent with prior literature.^1,4^ When comparing various counterfactuals, removal of the March–April 2020 period reduced estimates of excess HAIs. Thus, some of the initial increase in HAI rates during the pandemic may have been associated with the COVID-19 pandemic, although our statistical power was inadequate to characterize these effects precisely.

Reasons for the higher rates of HAIs during the pandemic period (compared to predicted rates in the counterfactual scenarios) may be multifactorial, including personal protective equipment shortages and changes in infection prevention practices (eg, placing patients in cohorts, changing contact precautions recommendations to preserve personal protective equipment, etc) as has been suggested previously.^16^ Other contributing factors may have included increased length of hospital stay, an overburdened healthcare system, high patient-to-staff ratios due to increased staffing issues, and staff burnout.^16^

Our study was limited by its regional nature, with all 3 hospitals located in the St. Louis metropolitan area, which may not be representative of patient populations and risk factors for HAIs across the state and country. In addition, because these are observational data, we cannot be certain that the results are a direct result of the pandemic and not some other confounding factor. To best address this, we included a dichotomous term for the pandemic and a separate term for rates of COVID-19 cases. Our study was limited by the amount of data available, which reduced our statistical power; thus, some of the null results we found are likely due to lack of power rather than lack of true effect. This factor can be explored further in future studies with larger cohorts.

Our study was strengthened by the rigorous analytical method utilized. We investigated counterfactual scenarios to estimate the effects of different aspects of the pandemic on the number of HAIs: one (ie, ‘no COVID-19 cases’) was intended to separate the effect of COVID-19 caseload from that of other pandemic-related disruptions, and a second (ie, ‘no pandemic’) was designed to assess the impact of the pandemic overall by generating predicted HAIs in a scenario without the pandemic. Importantly, these hypothetical HAIs under ‘no pandemic’ were based on forecasts of pre-existing trends in related covariates, rather than an assumption of a simple linear trend. To our knowledge, this is the first time this approach has been used to understand HAI rates during the COVID-19 pandemic.

In conclusion, the early COVID-19 era was variably associated with increases in CLABSIs, CAUTIs, and VAEs at 3 hospitals in a metropolitan region in Missouri, with variations in risk by hospital. Continued vigilance in maintaining optimal infection prevention practices to minimize HAIs is warranted, including hand hygiene, minimizing unnecessary device use, and promoting antimicrobial stewardship.

## Supplementary material

For supplementary material accompanying this paper visit https://doi.org/10.1017/ash.2022.361. click here to view supplementary material
