# Supplementary material for: Healthcare-associated infections (HAIs) during the coronavirus disease 2019 (COVID-19) pandemic: A time-series analysis
Source: Antimicrob Steward Healthc Epidemiol. 2023 Jan 17;3(1):e14. doi: 10.1017/ash.2022.361 (PMC9879893; doi:10.1017/ash.2022.361)
Supplement: Supplementary file 1 [file S2732494X22003618sup001.docx]

**Supplemental Table 1:** Actual and predicted HAIs under counterfactual scenarios

| Hospital | HAI type | Actual,  Mar–Aug 2020 | Actual, May–Aug 2020 | No Cases,  Mar–Aug 2020 | | No Cases,  May–Aug 2020 | | No Pandemic,  Mar–Aug 2020 | | No Pandemic,  May–Aug 2020 | |
| --- | --- | --- | --- | --- | --- | --- | --- | --- | --- | --- | --- |
|  |  | HAIs | HAIs | Predicted HAIs | Actual – Predicted | Predicted HAIs | Actual – Predicted | Predicted HAIs | Actual – Predicted | Predicted HAIs | Actual – Predicted |
| Hospital 1 | CLABSI | 78 | 63 | 51.7 (34.6, 77.8) | 26.3 (0.2, 43.4) | 54.7 (29.4, 102.0) | 8.3 (-39.0, 33.6) | 84.7 (62.5, 115.0) | -6.7 (-37.0, 15.5) | 56.4 (41.6, 76.4) | 6.6 (-13.4, 21.4) |
|  | CAUTI | 33 | 25 | 25.8 (14.5, 46.4) | 7.2 (-13.4, 18.5) | 14.6 (6.4, 33.2) | 10.4 (-8.2, 18.6) | 47.1 (32.0, 69.3) | -14.1 (-36.3, 1.0) | 30.6 (20.8, 45.0) | -5.6 (-20.0, 4.2) |
|  | VAE | 88 | 59 | 76.4 (49.9, 117.7) | 11.6 (-29.7, 38.1) | 44.0 (24.9, 77.8) | 15.0 (-18.8, 34.1) | 75.7 (52.9, 108.4) | 12.3 (-20.4, 35.1) | 50.9 (35.7, 72.7) | 8.1 (-13.7, 23.3) |
| Hospital 2 | CLABSI | 13 | 4 | 3.5 (1.9, 6.6) | 9.5 (6.4, 11.1) | 3.2 (1.5, 7.0) | 0.8 (-3.0, 2.5) | 5.4 (3.5, 8.4) | 7.6 (4.6, 9.5) | 3.8 (2.5, 5.8) | 0.2 (-1.8, 1.5) |
|  | CAUTI | 6 | 3 | 1.8 (0.7, 4.6) | 4.2 (1.4, 5.3) | 0.7 (0.2, 1.9) | 2.3 (1.1, 2.8) | 1.4 (0.7, 3.1) | 4.6 (2.9, 5.3) | 0.7 (0.3, 1.8) | 2.3 (1.2, 2.7) |
|  | VAE | 17 | 11 | 13.3 (7.2, 25.3) | 3.7 (-8.3, 9.8) | 6.4 (3.2, 13.0) | 4.6 (-2.0, 7.8) | 8.8 (5.7, 13.5) | 8.2 (3.5, 11.3) | 5.8 (3.7, 8.9) | 5.2 (2.1, 7.3) |
| Hospital 3 | CLABSI | 11 | 7 | 3.7 (2.3, 6.1) | 7.3 (4.9, 8.7) | 3.9 (1.9, 7.9) | 3.1 (-0.9, 5.1) | 5.7 (3.8, 8.6) | 5.3 (2.4, 7.2) | 3.8 (2.5, 5.6) | 3.2 (1.4, 4.5) |
|  | CAUTI | 8 | 7 | 4.6 (2.5, 8.6) | 3.4 (-0.6, 5.5) | 2.5 (1.0, 6.3) | 4.5 (0.7, 6.0) | 6.6 (4.3, 10.4) | 1.4 (-2.4, 3.7) | 4.4 (2.8, 6.9) | 2.6 (0.1, 4.2) |
|  | VAE | 11 | 7 | 8.4 (5.3, 13.3) | 2.6 (-2.3, 5.7) | 5.2 (2.7, 10.0) | 1.8 (-3.0, 4.3) | 7.2 (4.7, 10.9) | 3.8 (0.1, 6.3) | 4.6 (3.1, 7.1) | 2.4 (-0.1, 3.9) |

**Supplemental Figure 1:** Estimating CAUTIs by hospital during the COVID-19 pandemic under actual and counterfactual scenarios^a^

Time series data in black are raw data, the remaining lines are predicted values and are adjusted for covariates.

^a^Grey area for March-April 2020 period not included in secondary analysis.

**Supplemental Figure 2:** Estimating VAEs by hospital during the COVID-19 pandemic under actual and counterfactual scenarios^a^

Time series data in black are raw data, the remaining lines are predicted values and are adjusted for covariates.

^a^Grey area for March-April 2020 period not included in secondary analysis.

**Supplemental Figure 3:** Estimating total excess CAUTIs by hospital during the COVID-19 pandemic with counterfactual scenarios

**Supplemental Figure 4**: Estimating total excess VAEs by hospital during the COVID-19 pandemic with counterfactual scenarios
